# Bone marrow mesenchymal stem cell-derived exosomal microRNA-124-3p attenuates neurological damage in spinal cord ischemia-reperfusion injury by downregulating Ern1 and promoting M2 macrophage polarization

**DOI:** 10.1186/s13075-020-2146-x

**Published:** 2020-04-09

**Authors:** Ran Li, Kunchi Zhao, Qing Ruan, Chunyang Meng, Fei Yin

**Affiliations:** Department of Spine Surgery, China-Japan Union Hospital, Jilin University, No. 126, Xiantai Street, Changchun, 130033 Jilin Province People’s Republic of China

**Keywords:** Spinal cord ischemia-reperfusion injury, Exosome, MicroRNA-124-3p, Ern1, Macrophage M2

## Abstract

**Background:**

Spinal cord ischemia-reperfusion injury (SCIRI) often leads to neurological damage and mortality. In this regard, understanding the pathology of SCIRI and preventing its development are of great clinic value.

**Methods:**

Herein, we analyzed the role of bone marrow mesenchymal stem cell (BMMSC)-derived exosomal microRNA (miR)-124-3p in SCIRI. A SCIRI rat model was established, and the expression of Ern1 and M2 macrophage polarization markers (Arg1, Ym1, and Fizz) was determined using immunohistochemistry, immunofluorescence assay, RT-qPCR, and western blot analysis. Targeting relationship between miR-124-3p and Ern1 was predicted using bioinformatic analysis and verified by dual-luciferase reporter assay. Macrophages were co-cultured with miR-124-3p-containing BMMSC-derived exosomes. M2 macrophages were identified using flow cytometry, and the expression of Arg1, Ym1, and Fizz was determined. In addition, SCIRI rats were injected with miR-124-3p-containing exosomes, spinal cord cell apoptosis was observed using TUNEL assay, and the pathological condition was evaluated with H&E staining.

**Results:**

In SCIRI, Ern1 was highly expressed and M2 polarization markers were poorly expressed. Silencing Ern1 led to elevated expression of M2 polarization markers. MiR-124-3p targeted and negatively regulated Ern1. Exosomal miR-124-3p enhanced M2 polarization. Highly expressed exosomal miR-124-3p impeded cell apoptosis and attenuated SCIRI-induced tissue impairment and nerve injury. miR-124-3p from BMMSC-derived exosomes ameliorated SCIRI and its associated nerve injury through inhibiting Ern1 and promoting M2 polarization.

**Conclusion:**

In summary, exosomal miR-124-3p derived from BMMSCs attenuated nerve injury induced by SCIRI by regulating Ern1 and M2 macrophage polarization.

## Background

Spinal cord ischemia-reperfusion injury (SCIRI) often results from a blockade of the aorta for a certain period occurring during spinal operation or aortic aneurysm surgery and may eventually induce paralysis or paraplegia and neural dysfunction, which can cause great damage to individuals both physically and mentally [1]. As SCIRI induced neurological damage is largely irreversible with current treatment approaches, its prevention and management have been the focus of much research. Bone marrow mesenchymal stem cell (BMMSC) transplantation is one of the most promising therapies for SCIRI due to BMMSC-derived exosomes [2]. Mesenchymal stem cell (MSC)-derived exosomes are known to function as cellular regulators by transporting proteins, lipids, and RNAs [3]. MSC-derived exosomes have been extensively studied and investigated as a novel strategy in cell-free therapy [4]. However, the function of BMMSC-derived exosomes in SCIRI needs to be explored further.

Endoplasmic reticulum (ER) is involved in transporting proteins or lipids to the cell surface or organelles [5] and ER stress has been suggested to occur as a pathological consequence of spinal cord injury [6], suggesting a role of ER-relevant signaling in SCIRI pathogenesis. Endoplasmic reticulum to nucleus signaling 1 (Ern1) is a signaling enzyme and a crucial sensor of unfolded proteins in the ER [7]. In the current study, we decided to explore the role of Ern1 in the process of SCIRI.

M2 macrophage is known as the alternatively activated macrophage and its polarization is thought to be of therapeutic value; specifically, increasing M2 polarization is known to attenuate inflammation [8]. A role of macrophages in the pathology of spinal cord injury has been previously reported [9]. Meanwhile, the macrophage polarization from M1 phenotype confers protection against development of myocardial ischemia/reperfusion injury [10], though the function of M2 polarization in SCIRI pathology is not well investigated. M2 activation is also characterized by increased levels of M2 macrophage markers (Arg1, Ym1, and Fizz1) [11, 12].

MSC-secreted exosomes (usually sized 30–120 nm) not only mediate inter-cellular communication, but are also correlated with microRNAs (miRNAs, miRs) closely [13]. MiR-124 has been detected in the human and rat brain, and miR-124-3p, a member of the miR-124 family, is implicated in neurodegenerative disorders such as Parkinson’s disease [14]. However, a role for miR-124-3p in BMMSC-derived exosomes has not yet been illustrated.

In this study, we intended to explore the roles of exosomes, Ern1, and M2 macrophages in SCIRI. We hypothesized that miR-124-3p in BMMSC-derived exosomes could reduce neurological damage in SCIRI by depleting Ern1 to enhance M2 polarization. This pathway could emerge as possible target relieving SCIRI-related nerve injury.

## Methods

### Ethical statement

All animal experiments were approved by Animal Ethics Committee of China-Japan Union Hospital, Jilin University. The animals received humane care in accordance with the Guide for the Care and Use of Laboratory Animals published by the US National Institutes of Health and utmost care was taken to minimize the numbers and suffering of included animals.

### SCIRI animal model

Sixty-two SD rats (males; aged 8 weeks) were purchased from the Center of Laboratory Animals, Jilin University, China (license No. SCXK(Ji)2008-0005). A model of spinal cord ischemia/reperfusion injury was prepared as previously described [15]. In brief, rats were injected intraperitoneally with 10% chloral hydrate (3 mL/kg) and fixed lying on their side. A 5-cm incision was made down from the lower edge midline of the left ribs. The left kidney was located, followed by locating the abdominal aorta along the renal artery, which was ligated with a 10-g bulldog clamp placed below the renal artery for 1 h. The bulldog clamp was removed and the abdominal cavity closed after washing with penicillin. Rats in the sham surgery group only received laparotomy without ligation of the abdominal aorta. The model was deemed as successfully established if neurological deficits appeared in the hindlimb. Controls were not given any treatment. The rats were euthanized after completion of the experiment and the spinal cord (L2–5) tissue was collected for subsequent analysis.

### Isolation, culture, and identification of macrophages

The spinal cord was washed using phosphate buffer saline (PBS), filtered using a 100-μM cell filter and centrifuged at 1200 rpm for 5 min. Next, collected cells were cultured in the RPMI-1640 medium containing 10 μg/mL monocyte colony-stimulating factor, 100 U/mL penicillin, and 100 μg/mL streptomycin at 37 °C in 5% CO_2_ for 6–8 days. Macrophage markers (F4/80 or CD11b) were identified using a flow cytometer (FA CSCalibur, BD Biosciences, San Jose, CA, USA).

### Isolation and culture of BMMSCs

Six rats were euthanized using the same method as described above. After the rats were immersed in 75% ethyl alcohol for 5 min, the leg bone on both sides was extracted and placed into a medium containing PBS for removal of the muscle tissue attached to the bone. The bone was then placed in a plate containing pre-cold Dulbecco’s modified Eagle’s medium (DMEM) with 10% fetal bovine serum (FBS), 100 U/mL penicillin sodium, and 0.1 g/L streptomycin. The bone marrow cavity was repeatedly washed with the culture solution, which was then collected. Cells in the solution were repeatedly triturated and rested for 10 min, and the supernatant was transferred into a 10-mL sterile centrifugal tube and centrifuged at 3000 rpm at 4 °C for 3 min. Cells were resuspended in L-DMEM with 10% FBS and then incubated in a 25-cm^2^ culture dish at a density of 5 × 10^5^ cells/mL at 37 °C with 5% CO_2_ and 95% humidity. After 24 h, non-adherent cells were removed and morphology of the cells was observed.

The 4th and the 8th generation of cultured cells were collected and centrifuged at 1000 rpm at 4 °C for 5 min. The cells (5 × 10^5^ cells/tube) were incubated with diluted monoclonal antibody against CD29 (1:200; ab179471), CD44 (1:200; ab157107), CD34 (1:100; ab81289), and CD45 (1:100; ab10558, all from Abcam Inc., Cambridge, UK) at room temperature for 30 min. Last, cells were analyzed by flow cytometry.

### Extraction and identification of exosomes derived from BMMSCs

The BMMSCs were cultured in DMEM with exosome-free FBS (Hyclone, South Logan, UT, USA). The culture medium was collected, filtered using a 0.22-μM cell filter, and then concentrated using ultrafiltration (Millipore Corp., Bedford, MA, USA). Exosomes were obtained following the manufacturer’s protocol of exosome isolation kit (Invitrogen, Carlsbad, CA, USA). A total of 10 μL extracted exosomes were diluted with equal volume of PBS and then negatively stained using 3% sodium phosphotungstate solution for 1 min. After washing with distilled deionized water and drying at room temperature, the exosomes were observed and photographed under a transmission electron microscope (Hitachi, Tokyo, Japan).

Dynamic light scattering was performed to measure exosome particle diameter. Particle size distribution was analyzed using Zetasizer Nano ZS90 (Malvern Panalytical, UK). The diluted samples in PBS in the ratio of 1:20 were manually loaded into the sample chamber. Three videos (60 s) were recorded of each sample. Data was analyzed using DTS v5.10 software (Malvern Panalytical, UK).

### Animal treatment

A total of 50 rats were used for the study with 10 rats in each group: control group (without artery occlusion), sham group (sham-operated rats without artery occlusion), SCRI group (untreated SCRI rat model), SCIRI-Bmexos-mimic NC group (SCRI rat model injected with mimic NC exosomes), and SCIRI-Bmexos-miR-124-3p mimic group (SCRI rat model injected with miR-124-3p mimic exosomes). Caudal intravenous injections of exosomes were performed at 5 × 10^10^ particles/100 μL, and equal volumes of PBS were injected in control rats.

### Plasmid transfection

Macrophages derived from rat bone marrow were seeded into a six-well plate at a density of 4 × 10^5^ cells/well, and transfected according to the manufacturer’s instruction of Lipofectamine 2000 (11668-019, Invitrogen, Carlsbad, CA, USA). Cells were transfected with miR-124-3p mimic, miR-124-3p inhibitor, shRNA against Ern1, or their relevant NC individually or together at the final concentration of 50 nM.

### Co-culture of BMMSCs and macrophages

To prevent the release of exosomes from BMMSCs, BMMSCs were treated with exosome inhibitor GW4869. In brief, BMMSCs were seeded in a six-well plate at the density of 1 × 10^6^ cells/well and treated with 10% GW4869 (D1692-5MG, Sigma-Aldrich, St. Louis, MO, USA). Cells and supernatant were collected after 24-h treatment and used for further analyses. Whether exosome secretion in BMMSCs treated with inhibitor GW4869 was occluded or not was ascertained by observation under an electron microscope.

Thereafter, BMMSCs were inoculated in the basolateral chamber of a 24-well transwell at 1 × 10^4^ cells/well, while macrophages were seeded in the apical chamber. After 24-h co-culture, macrophages were collected. The expression of miR-124-3p and Ern1 was determined. BMMSCs were transfected with GW4869 (at the final concentration of 10 nM), or macrophages treated with miR-124-3p mimic, miR-124-3p inhibitor, or their relevant controls (at the final concentration of 50 nM).

Next, exosomes extracted from these BMMSCs were labeled by PKH67 (Green) staining solution (MINI67-1KT, Sigma-Aldrich, St. Louis, MO, USA) and co-cultured with macrophages for 48 h. The exosomes at a concentration of 100 μg/mL were adopted to treat cells in vitro. Thereafter, the expression of miR-124-3p, Ern1, Arg1, Ym1, and Fizz was determined by RT-qPCR and western blot analysis.

### Evaluation of hind-limb motor function

After 6 h of reperfusion, rats were placed within an enclosed annular metallic shell. The movements of rat hindlimbs were evaluated for 5 min by two examiners standing on the opposite side. The Basso Beattie Bresnahan scale was used to score and record the hindlimb motor nerve function.

### TUNEL assay

After the rats were euthanized, the spinal cord tissue was extracted, embedded, and sectioned. Each dewaxed section was incubated with 50 μL diluted proteinase K at 37 °C for 30 min, which was then fixed with 4% paraformaldehyde for 30 min. The sections were fixed in 0.3% H_2_O_2_-formaldehyde solution for 30 min and incubated with Triton X-100 (0.3%) on ice for 2 min. Next, the TUNEL reaction mixture was prepared in accordance with instructions of the TUNEL apoptosis detection kit (green fluorescence, C1088, Beyotime Biotechnology, Shanghai, China). A total of 50 μL terminal deoxynu-cleotidyl trans/erase was mixed with 450 μL fluorescein-labeled deoxyuridine triphosphate (dUTP). The sections were incubated with TUNEL reaction mixture. An apoptosis index was calculated as the percentage of positive cells observed under the fluorescence microscope.

### Blood-spinal cord barrier (BSCB) integrity detected by Evans blue (EB) staining

A caudal intravenous injection of 2% EB was performed 6 h after reperfusion injury on the rats. After 3 h, the rats were euthanized. The partial spinal cord (T_0−_T_11_) was removed and weighed. Then, the spine cord was immersed into 50% trichloroacetic acid for 3 days and centrifuged at 10,000*g* for 10 min, followed by removal of the supernatant. Optical density value was measured using a fluorescence microplate reader with an excitation wavelength of 620 nm and emission wavelength of 680 nm (BioTek Winooski, Vermont, USA). Lastly, spinal cord sections of 20 μM were observed using a fluorescence microscope, and the relative fluorescence intensity was calculated using Image Pro Plus 7.0 for qualitative analysis.

### Hematoxylin and eosin (H&E) staining

Spinal cord tissues extracted from euthanized rats were embedded, sectioned, and then fixed by 10% neutral formaldehyde solution over 24 h. Next, paraffin-embedded sections were dewaxed with dimethyl benzene, dehydrated with graded ethanol, and washed with distilled water. H&E staining was performed, followed by dehydration, and transparentized. After the section was dried, histological and morphological changes were observed using an optical microscope.

### Immunohistochemistry

Samples were fixed with 10% formalin, embedded by paraffin, and sectioned into 4 μM sections. The sections were dewaxed with dimethyl benzene and dehydrated with graded ethanol, followed by incubation in 3% H_2_O_2_ (Sigma-Aldrich, St. Louis, MO, USA) at 37 °C for 30 min and boiling in 0.01 M citric acid buffer at 95 °C for 20 min. The sections were then blocked with normal serum working solution at 37 °C for 10 min and incubated with diluted primary antibodies rabbit against Ern1 (1:200, ab48187), Arg1 (1:400, ab91279), Ym1 (1:200, ab192029), and Fizz (1:100, ab11429, all from Abcam Inc.) at 37 °C for 2 h. Then, the sections are incubated with Horseradish Peroxidase (HRP) conjugated secondary goat anti-rabbit immunoglobulin G (IgG) antibody and counterstained with hematoxylin (Chemcd, Shanghai, China) at room temperature for 4 min. Samples were mounted with 10% glycerinum/PBS and observed using microscope.

### Immunofluorescence assay

Cells were fixed with 4% paraformaldehyde and penetrated with 0.5% Triton X-100 (Sangon Biotech, Shanghai, China) at room temperature for 20 min. After washing with PBS, the cells were blocked with normal goat serum (Solarbio, Beijing, China) at room temperature for 30 min. Next, the cells were incubated with primary rabbit antibodies (same antibodies as we utilized in immunohistochemistry), washed with phosphate buffered solution (PBST), and incubated with diluted Alexa Fluor 647 conjugated donkey anti-rabbit IgG antibody (1:400, ab150075, Abcam Inc., Cambridge, UK) at 37 °C for 1 h. Then, the cells were incubated with 4′6-diamidino-2-phenylindole in the dark for 5 min, mounted with anti-fade mounting medium, and observed using a fluorescence microscope (Olympus, Tokyo, Japan).

### RNA isolation and quantitation

Total RNA was extracted using RNeasy Mini Kit (Qiagen, Valencia, CA, USA) and reverse transcribed into complementary DNA according to the manufacturer’s protocol of the reverse transcription kit (RR047A, Takara, Tokyo, Japan). qPCR was carried out following the instructions provided by the SYBR Premix EX Taq kit (RR420A, Takara, Tokyo, Japan) using a real-time fluorescence quantitative PCR appliance (ABI7500, Applied Biosystems, Foster City, CA, USA). All primers were synthesized by Sangon Biotech (Shanghai, China) (Table 1). The relative expression of the target gene was calculated using the 2^−ΔΔCt^ method where GAPDH and U6 were taken as internal reference genes.

**Table 1:** Primer sequence for RT-qPCR

Genes	Primer sequence
Ern1	F: 5′-GAGATGTGGCCCTGAAACCT-3′
	R: 5′-AGCAAGCTCATCCGGTGAAA-3′
MiR-124-3p	F: 5′-ATGTTCACAGCGGACCTTGAT-3′
	R: 5′-TTCACCGCGTGCCTTAATTG-3′
Arg1	F: 5′-GGAAATCGTGGAAATGAG-3′
	R: 5′-CAGATATGCAGGGAGTCACC-3′
Ym1	F: 5′-TTCTTGTCACAGGTCTGG-3′
	R: 5′-CCTTAGCCCAACTGGTATAGT-3′
Fizz	F: 5′-CAGGATGCCAACTTTGAATAGG-3′
	R: 5′-CACAAGCACACCCAGTAGCAGTC-3′
GAPDH	F: 5′-CCTTCATTGACCTCAACTAC-3′
	R: 5′-GGAAGGCCATGCCAGTGAGC-3′
U6	F: 5′-GGTCGGGCAGGAAAGAGGGC-3′
	R: 5′-GCTAATCTTCTCTGTATCGTTCC-3′

Note: *Ern1* endoplasmic reticulum to nucleus signaling 1, *MiR-124-3p* microRNA-124-3p, *Arg1* arginase 1, *Ym1* chitinase-like 3; *Fizz*, *GAPDH* glyceraldehyde-3-phosphate dehydrogenase

### Western blot analysis

Total protein from tissues or cells was isolated by radioimmunoprecipitation lysis buffer containing phenylmethylsulphonyl fluoride. Next, 50 μG protein was separated by sodium dodecyl sulfate-polyacrylamide gel electrophoresis and transferred onto a polyvinylidene fluoride (PVDF) membrane, which was blocked with 5% skim milk at room temperature for 1 h. The PVDF membrane was then incubated with diluted primary rabbit antibodies against mouse CD63 (Abcam; ab217345, 1:1000), TSG101 (Abcam; ab125011, 1:1000), calnexin (Abcam: ab22595, 1:1000), Ern1 (1:200, ab37073), Arg1 (1:400, ab91279), Ym1 (1:200, ab192029), Fizz (1:100, ab11429), and GAPDH (1:2500, ab9485) overnight at 4 °C. After washing with Tris Buffered Saline with Tween, the membrane was incubated with HRP-labeled secondary goat anti-rabbit antibody IgG (1:2000, ab97051, Abcam Inc.) for 1 h. Next, the membrane was developed using ECL detection reagent (Thermo Fisher Scientific, Waltham, MA, USA), which was photographed by Bio-Rad Image analysis system (Hercules, CA, USA) and analyzed using Quantity One v4.6.2 software. The relative protein level was quantified as the gray value of target protein band/GAPDH.

### Flow cytometry

Cluster of differentiation (CD) 68, CD86, and CD163 antibodies were used to identify the subtype of macrophages through flow cytometry. The cells were incubated with the following monoclonal antibody: Fluorescein isothiocyanate conjugated antibody against CD68 (1 μG/10^6^ cells, ABD Serotec, Kidlington, Oxford, UK), Alexa Fluor 647 conjugated CD163 antibody (1 μG/10^6^ cells, ABD Serotec), and PE conjugated CD86 antibody (0.2 μG/10^6^ cells, eBioscience, San Diego, CA, USA). After washing with staining buffer solution, cells were fixed with 1% paraformaldehyde and analyzed using a FACSCalibur flow cytometer (Becton Dickinson, San Diego, CA, USA). Then, a minimum of 10,000 cells were collected and analyzed using WinMDI 2.8 software (J. Trotter, The Scripps Research Institute, La Jolla, CA, USA).

### Dual-luciferase reporter assay

Ern1 wild-type (WT) containing miR-124-3p binding sites on the Ern1 promoter region and Ern1 mutant type (MUT) in which the miR-124-3p binding sites were mutated, were ligated into PmirGLO vectors, respectively. Either PmirGLO-Ern1-WT or PmirGLO-Ern1-MUT was co-transfected with miR-124-3p mimic or NC mimic plasmid into HEK293T cells. After 24 h, the cells were collected and lysed. Luciferase activity was detected using a Dual-Luciferase® Reporter Assay System (E1910, Promega, Madison, WI, USA). The relative luciferase activity was expressed as the ratio of firefly luciferase activity to renilla luciferase activity.

### Statistical analysis

All data analyses were performed using SPSS 21.0 (IBM Corp, Armonk, NY, USA). The measurement data were expressed as mean ± standard deviation. Differences between two unpaired groups that were normally distributed and with uniform variance were analyzed using unpaired *t*-test, while those between multiple groups were analyzed using one-way analysis of variance, followed by a Turkey’s post hoc test. Values of *p* < 0.05 indicated statistical significance.

## Results

### Ern1 is highly expressed and polarization of M2 macrophage is attenuated in SCIRI

**Fig. 1:**
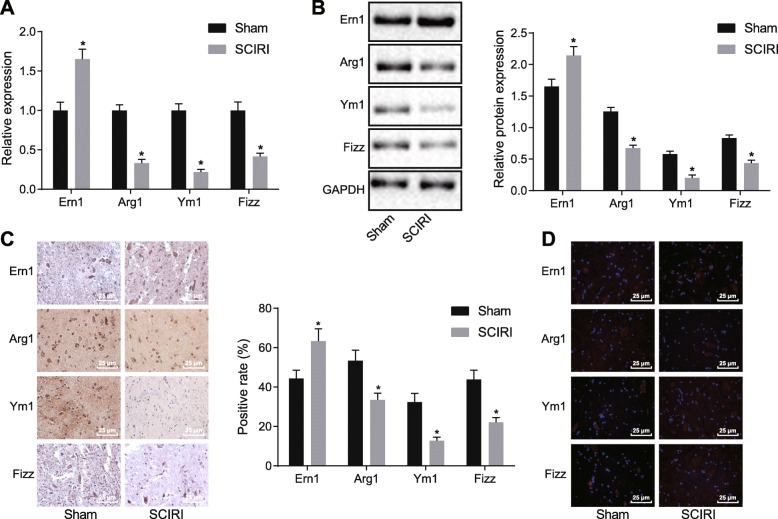
Increased Ern1 and inhibited M2 macrophage polarization in SCIRI rats. **a** Expression of Ern1, Arg1, Ym1, and Fizz in SCIRI rats detected by RT-qPCR; **b** Ern1, Arg1, Ym1, and Fizz expression in SCIRI rats determined by western blot analysis; **c** Ern1, Arg1, Ym1, and Fizz expression in SCIRI rats measured by immunohistochemistry (× 400); **d** Ern1, Arg1, Ym1, and Fizz expression in SCIRI rats examined by immunofluorescence assay (× 400). *p < 0.05 versus sham operation. Experimental data were measurement data and expressed as mean ± standard deviation. Difference between 2 experimental groups was analyzed by unpaired *t-*test; *n* = 10

In an attempt to observe Ern1 expression and polarization of M2 macrophage in SCIRI, an SCIRI rat model was established. Expression of Ern1 as well as M2 macrophage polarization markers including Arg1, Ym1, and Fizz in SCIRI rats was detected by RT-qPCR (Fig. 1a). It was revealed that as compared with normal rats, in SCIRI rats, Ern1 expression was elevated, while expression of Arg1, Ym1, and Fizz was significantly declined (*p* < 0.05). The expression of Ern1, Arg1, Ym1, and Fizz was also determined by western blot analysis (Fig. 1b), showing that in contrast to normal rats, the protein level of Ern1 was significantly increased, while the protein levels of Arg1, Fizz, and Ym1 were decreased in SCIRI rats (*p* < 0.05). Immunohistochemistry and immunofluorescence assay (Fig. 1c, d) also illustrated that Ern1 expression was enhanced, while Arg1, Ym1, and Fizz expression was attenuated in SCIRI rats (*p* < 0.05). In conclusion, it was suggested that Ern1 expression was increased and M2 macrophage polarization was reduced in SCIRI rats.

### Ern1 regulates polarization of M2 macrophages

**Fig. 2:**
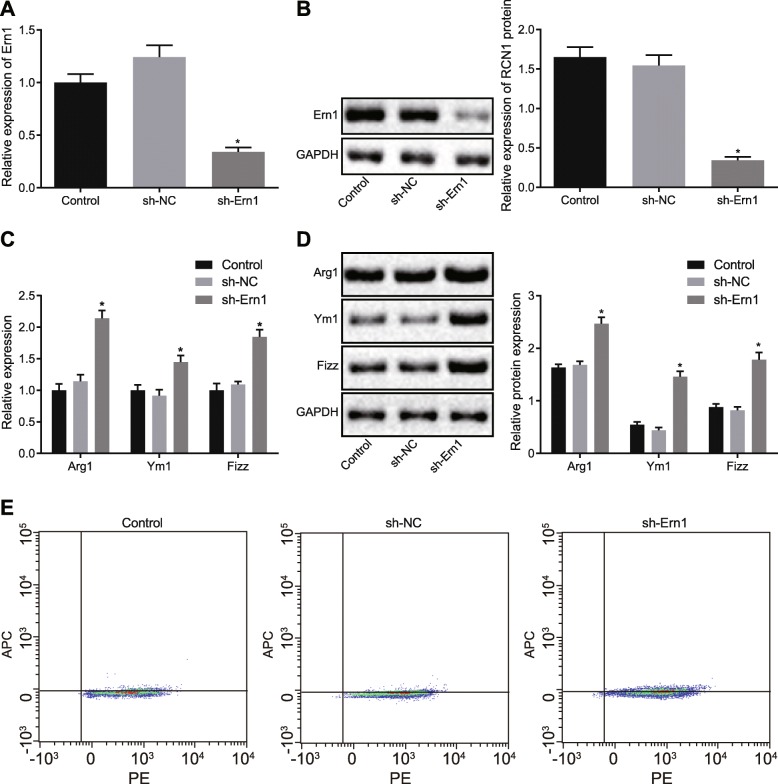
Macrophage polarization was modulated by Ern1. **a** Ern1 expression in macrophages transfected with different plasmids detected by RT-qPCR. **b** Ern1 expression in macrophages transfected with different plasmids determined by western blot analysis. **c** Arg1, Ym1, and Fizz expression in differently treated macrophages measured by RT-qPCR. **d** Arg1, Ym1, and Fizz expression in differently treated macrophages examined by western blot analysis. **e** M2 macrophage proportion in differently treated macrophages identified by flow cytometry. *p < 0.05 versus control. The measurement data were presented as mean ± standard deviation. One-way ANOVA was applied to compare differences between multiple groups, followed by a Tukey’s post hoc test. The cell experiment was repeated thrice

To investigate the regulatory role of Ern1 in M2 macrophage polarization, macrophages were transfected with shRNA targeting Ern1. Ern1 expression was detected using RT-qPCR and western blot analysis (Fig. 2a, b), suggesting that compared with shRNA against NC, Ern1 expression was distinctly reduced in macrophages transfected with shRNA against Ern1 (*p* < 0.05). The expression of Arg1, Ym1, and Fizz was determined by RT-qPCR and western blot analysis (Fig. 2c, d), which showed that silencing Ern1 significantly increased the expression of Arg1, Ym1 and Fizz (*p* < 0.05). Furthermore, the proportion of M2 macrophages was identified using a flow cytometer (Fig. 2e). Compared with shRNA against NC, shRNA against Ern1 enhanced the proportion of M2 macrophage polarization (*p* < 0.05). These results demonstrated that Ern1 was able to negatively mediate polarization of M2 macrophages.

### MiR-124-3p targets Ern1

**Fig. 3:**
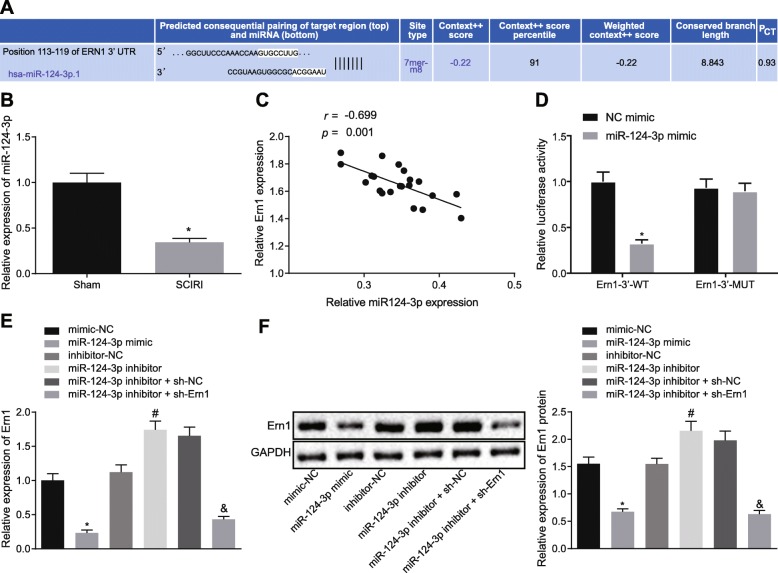
Ern1 was targeted by miR-124-3p. **a** Possible target genes of miR-124-3p predicted by bioinformatic analysis. **b** Expression of miR-124-3p in SCIRI tissues detected by RT-qPCR, *p < 0.05 versus sham operation. **c** Correlation between miR-124-3p and Ern1 expression in spinal cord tissues. **d** Dual-luciferase activity detection. **e** Ern1 expression in macrophage cells transfected with different plasmids determined by RT-qPCR. **f** Ern1 expression in differently treated macrophages measured by western blot analysis. Difference between two experimental groups conforming to normal distribution was analyzed by unpaired *t*-test. Comparison between two groups was analyzed by independent-sample *t*-test. Correlation between *X*-axis and *Y*-axis values was analyzed by Pearson’s correlation coefficient. In Fig. **d**, *p < 0.05 versus NC mimic. In **e** and **f**, *p < 0.05 versus NC mimic; #*p* < 0.05 versus inhibitor treatment; &*p* < 0.05 versus miR-124-3p inhibitor with shRNA versus NC. The measurement data were expressed as mean ± standard deviation. Unpaired *t*-test was used to compare two experimental groups (*n* = 10); one-way ANOVA was utilized to analyze differences between multiple groups with Tukey’s post hoc test; correlation between miR-124-3p and Ern1 was analyzed by Pearson’s correlation coefficient. The experiment was repeated three times

To explore how Ern1 was regulated in SCIRI, putative upstream miRNAs that can regulate Ern1 were explored, and miR-124-3p was identified as one of these (Fig. 3a). Thereafter, the targeting relationship between miR-124-3p and Ern1 was confirmed by dual-luciferase reporter assay (Fig. 3d), which displayed that compared with NC mimic, the luciferase activity of Ern1-WT was significantly lowered upon co-transfection with miR-124-3p mimic (*p* < 0.05), while luciferase activity of Ern1-MUT showed no significant change (*p* > 0.05). The results illustrated that miR-124-3p could target Ern1.

Then, RT-qPCR (Fig. 3b) showed that miR-124-3p was poorly expressed in SCIRI rats (*p* < 0.05). Next, the correlation between the expression of miR-124-3p and Ern1 in spinal cord tissues (*n* = 21) was analyzed using RT-qPCR (Fig. 3c), which suggested that miR-124-3p was negatively correlated with Ern1. Furthermore, macrophages were transfected with miR-124-3p mimic and Ern1 expression was quantified using RT-qPCR and western blot analysis (Fig. 3e, f). The results showed that Ern1 expression was reduced by miR-124-3p mimic (*p* < 0.05). Together, these results evidenced that macrophage miR-124-3p targeted and negatively regulated Ern1 expression.

### Culture and identification of BMMSCs and isolation of exosomes

**Fig. 4:**
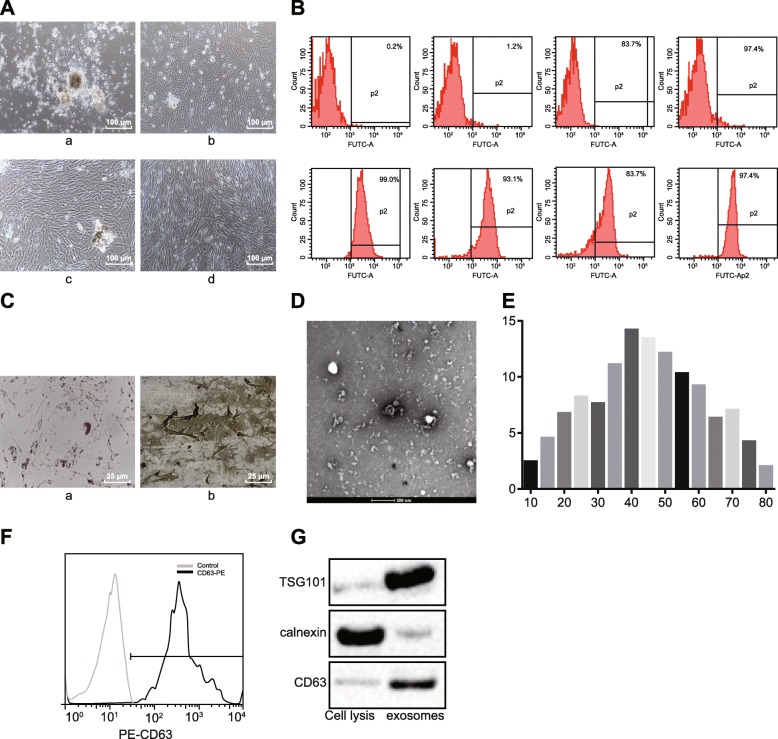
Separation and identification of BMMSCs and exosomes. **A** Morphological characteristics of BMMSCs (× 100); (a) primary cells; (b) cells after 3-day culture; (c) cells after 12-day culture; (d) P3 generation cells. **B** Surface markers of BMMSCs determined by flow cytometry. **C** Osteogenesis- and adipogenesis-inducing cultures of BMMSCs; (a) osteogenic differentiation, (b) adipogenic differentiation (× 400). **D** Exosomes identified by transmission electron microscope (× 50,000). **E** Diameter distribution of BMMSC-derived exosomes measured by Image-Pro plus software. **F** Expression of exosomal surface marker CD63 determined by flow cytometry. **G** Levels of CD63, TSG101, and calnexin examined by western blot analysis. *p < 0.05 versus cell lysis buffer. The measurement data were described as mean ± standard deviation. Independent-sample *t*-test was employed to analyze difference between two groups. The cell experiment was repeated three times

As a previous study reported that exosomes derived from BMMSCs played a significant role in SCIRI [2], the role of these exosomes was further investigated by isolating BMMSCs and extracting exosomes. The BMMSCs showed typical cellular characteristics 3 days after culture (Fig. 4A). The BMMSC antigen expression was analyzed by flow cytometry (Fig. 4B), displaying that CD90 positive-cells accounted for 90.0% of the total cells, and CD34-, CD31-, and CD45-positive cells accounted for less than 3% of the total cells, which aligned with established biological characteristics of BMMSCs. Osteogenic and adipogenic differentiation of BMMSCs were performed (Fig. 4C), which showed that after a 21-day osteogenesis-inducing culture, the cells were aligned in a multilayer and overlapped manner with calcium nodule formation, suggesting that the isolated BMMSCs had osteogenic differentiation potential (Fig. 4C, a). After a 25-day adipogenesis-inducing culture, the cells displayed characteristics of adipocytes, suggesting a potential for adipogenic differentiation (Fig. 4C-b).

Next, exosomes derived from BMMSCs were observed under a transmission electron microscope. The size of the exosomes ranged from 30 to 120 nm. Exosomes were either spherical or oval membrane vesicles (Fig. 4D). Exosome particle diameter was measured by dynamic light scattering (Fig. 4E). The expression of the exosomal surface marker CD63 was examined using flow cytometry (Fig. 4F), which demonstrated its elevated expression. As reflected in Fig. 4G, the level of CD63 and TSG101 expression was potently increased in the absence of calnexin protein in the exosomal lysate compared with cell lysate. These results confirmed the successful isolation of BMMSCs and exosome extraction.

### MiR-124-3p contained in BMMSC-derived exosomes regulates Ern1 expression in macrophage

**Fig. 5:**
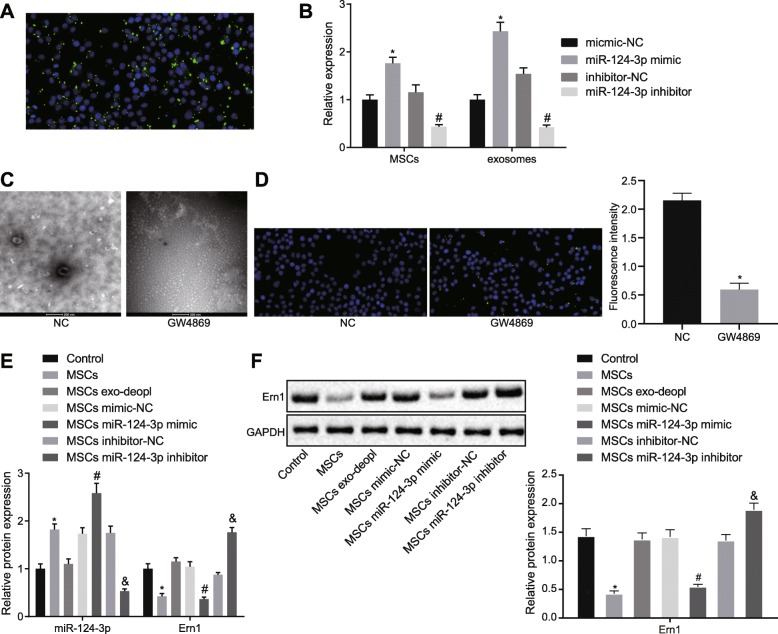
Expression of Ern1 in macrophages was mediated by exosomal miR-124-3p derived from BMMSCs. **a** Macrophages ingesting exosomes observed by confocal fluorescence microscope (× 200). **b** Expression of miR-124-3p in BMMSCs and exosomes detected by RT-qPCR; *p < 0.05 versus mimic NC; #*p* < 0.05 versus inhibitor NC. **c** Exosome secretion observed by transmission electron microscope (× 50,000). **d** The fluorescence PKH67 (Green) after GW4869 treatment observed by confocal fluorescence microscope. **e** Expression of miR-124-3p and Ern1 in co-cultured macrophages detected by RT-qPCR. **f** Expression of miR-124-3p and Ern1 in co-cultured macrophages determined by western blot analysis. In **d** and **e**, *p < 0.05 versus control; #*p* < 0.05 versus NC of BMMSCs mimic; &*p* < 0.05 versus NC of BMMSCs inhibitor. The data in figures were measurement data and described as mean ± standard deviation. Comparison between two groups was performed using unpaired *t*-test. Multiple-group comparison was made using one-way ANOVA, followed by a Tukey’s post hoc test. The cell experiment was repeated three times

In order to determine how the expression of Ern1 in macrophages was regulated, exosomes traced by PKH67 (Green) were co-cultured with macrophages. Macrophages ingesting the exosomes were observed under a confocal fluorescence microscope at the 3rd, 6th, and 12th hour. Figure 5a shows that the longer the co-culture period, the more macrophages displayed red fluorescence, which indicated that an increasing number of exosomes traced by PKH67 was ingested by the macrophages over time. To further demonstrate that exosomes released from BMMSCs could transfer miR-124-3p to macrophages, miR-124-3p expression in BMMSCs was altered. After increasing or decreasing miR-124-3p expression in BMMSCs, miR-124-3p expression in exosomes derived from these BMMSCs and macrophages was determined by RT-qPCR (Fig. 5b). The results displayed that miR-124-3p expression in exosomes and macrophages was significantly increased when BMMSCs were treated with miR-124-3p mimic (*p* < 0.05), but significantly declined when BMMSCs were treated with miR-124-3p inhibitor (*p* < 0.05).

To confirm the miR-124-3p was transferred into macrophages by the exosomes, the exosome inhibitor GW4869 was employed to inhibit exosome secretion from BMMSCs, which were then observed under an electron microscope (Fig. 5c). In addition, GW4869-treated exosomes were co-cultured with cells and observed under a fluorescence microscope, which revealed that the fluorescence significantly decreased after GW4869 treatment, suggesting the secretion of exosomes was markedly blocked by GW4869 treatment (Fig. 5d). Then, the expression of miR-124-3p and Ern1 in macrophages was determined using RT-qPCR and western blot analysis after co-culture of GW4869-treated BMMSCs and macrophage (Fig. 5e, f). Ern1 expression was decreased and miR-124-3p expression was enhanced in macrophages co-cultured with BMMSCs overexpressing miR-124-3p (*p* < 0.05), which was blocked when BMMSCs were treated with GW4869. These results illustrated that miR-124-3p transferred by exosomes effectively inhibited Ern1 expression in macrophages and suggested the crucial role of exosomes in the process of transfer of miRNA from BMMSCs to macrophages. In sum, BMMSCs regulated the expression of Ern1 in macrophages via exosomes containing miR-124-3p.

### MiR-124-3p in exosomes mediates Ern1 expression to induce M2 polarization of macrophages

**Fig. 6:**
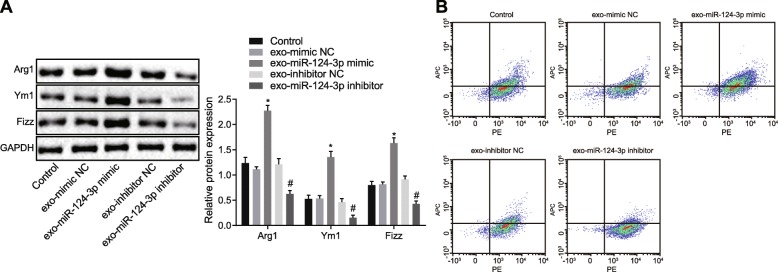
M2 macrophage polarization was induced by miR-124-3p via regulation of Ern1. **a** Markers of M2 macrophage polarization (Arg1, Ym1, and Fizz) expression determined by western blot analysis; **a** *p < 0.05 versus NC of exosome mimic; #*p* < 0.05 versus NC of exosome inhibitor. **b** M2 macrophages identified using flow cytometry. Measurement data were expressed as mean ± standard deviation. One-way ANOVA was used to analyze the difference between multiple groups with Tukey’s post hoc test. The cell experiment was repeated three times

Since exosomes derived from BMMSCs transferred miR-124-3p to macrophages and regulated Ern1 expression in macrophages, we explored whether M2 macrophage polarization was affected by this event. Exosomes isolated from BMMSCs transfected with various plasmids were co-cultured with macrophages, and M2 macrophage polarization was detected. Expression of Arg1, Ym1, and Fizz was determined by western blot analysis and immunofluorescence assay. According to Fig. 6a, Arg1, Ym1, and Fizz expression was distinctly elevated in macrophages co-cultured with exosomes with miR-124-3p mimic (*p* < 0.05). M2 macrophage was identified using flow cytometry (Fig. 6b), which exhibited that the M2 macrophage proportion was significantly elevated upon co-culture with exosomes with miR-124-3p mimic (*p* < 0.05). These results comprehensively demonstrated that exosomes derived from BMMSCs containing miR-124-3p mediated Ern1 expression in macrophages, therefore inducing polarization to M2 macrophage phenotype.

### Exosome derived from BMMSCs carrying miR-124-3p modulates Ern1 and augments polarization of M2 macrophage in vivo

**Fig. 7:**
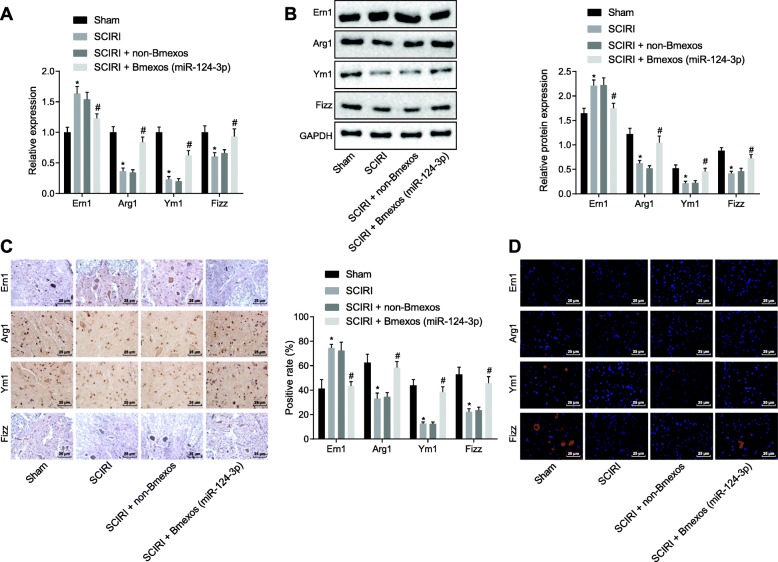
In vivo experiment demonstrating exosomal miR-124-3p derived from BMMSCs regulated Ern1 and polarization of M2 macrophage in SCIRI rats. **a** Ern1, Arg1, Ym1, and Fizz expression in rats spinal cord tissues determined using RT-qPCR. **b** Ern1, Arg1, Ym1, and Fizz protein levels in rat spinal cord tissues measured by western blot analysis. **c** Immunohistochemistry assay of Ern1, Arg1, Ym1, and Fizz expression in rat spinal cord tissues (× 400). **d** Immunofluorescence assay of Ern1, Arg1, Ym1, and Fizz expression in rats spinal cord tissues (× 400). *p < 0.05 versus sham operation; #*p* < 0.05 versus SCIRI treatment without exosome derived from BMMSCs. All measurement data were described as mean ± standard deviation. Comparison between two groups was conducted using unpaired *t*-test; *n* = 10

With an aim to further verify the regulation of miR-124-3p on Ern1 and M2 macrophage polarization in SCIRI in vivo*,* SCIRI rats were injected with exosomes derived from BMMSCs carrying miR-124-3p. The expression of Ern1, Arg1, Ym1, and Fizz in rats was determined by RT-qPCR and western blot analysis (Fig. 7a, b), demonstrating that in spinal cord tissues from SCIRI rats, Ern1 expression was markedly increased (*p* < 0.05), and Arg1, Ym1, and Fizz expression was reduced (*p* < 0.05), which could all be blocked by injection with exosomes carrying miR-124-3p. The above expression patterns of Ern1, Arg1, Ym1, and Fizz were also confirmed by immunohistochemistry and immunofluorescence assays (Fig. 7c, d). All these results demonstrated that exosomes derived from BMMSCs carrying miR-124-3p promoted polarization of M2 macrophage by regulating Ern1 expression in SCIRI in vivo.

### MiR-124-3p ameliorates SCIRI

**Fig. 8:**
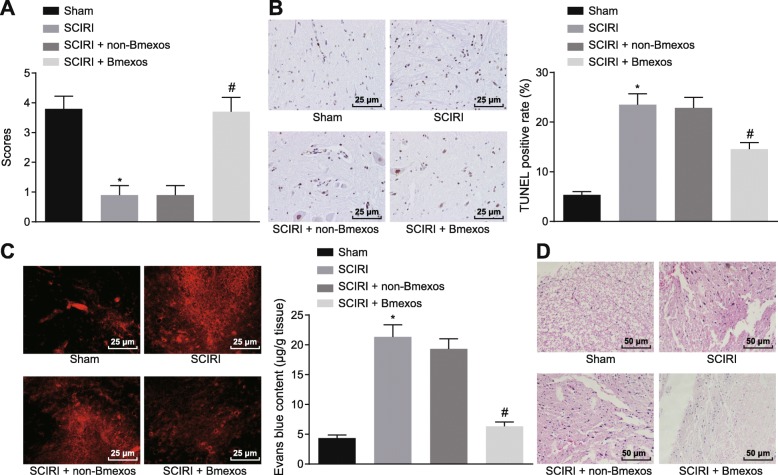
SCIRI could be alleviated by miR-124-3p overexpression. **a** Evaluation of hindlimb motor function in rats. **b** Cell apoptosis in spinal cord tissues determined by TUNEL assay (× 400). **c** BSCB integrity tested by EB staining (× 400). **d** H&E staining of spinal cord tissues of rats (× 200). *p < 0.05 versus sham operation; #*p* < 0.05 versus SCIRI rats without exosomes derived from BMMSCs. Measurement data were described as mean ± standard deviation. Unpaired *t-*test was used to compare the difference between two experimental groups; *n* = 10

In order to study the role of miR-124-3p in SCIRI, rat hind-limb motor function was evaluated (Fig. 8a). SCIRI rats with exosomes derived from BMMSCs carrying miR-124-3p scored much higher than untreated SCIRI rats. Spinal cord cell apoptosis was examined using TUNEL assay (Fig. 8b), which showed that cell apoptosis was significantly increased in SCIRI rats with exosomes derived from BMMSCs carrying miR-124-3p compared with untreated SCIRI rats (*p* < 0.05). BSCB integrity was then detected by EB staining (Fig. 8c), displaying that the BSCB integrity degree was significantly higher in SCIRI rats with exosomes derived from BMMSCs carrying miR-124-3p than that in untreated SCIRI rats (*p* < 0.05). The SCIRI degree was observed by H&E staining (Fig. 8d), which demonstrated that in contrast to untreated SCIRI rats, fewer vacuoles and lower-degree injury were found in SCIRI tissues with exosomes derived from BMMSCs carrying miR-124-3p. In conclusion, these results illustrated that miR-124-3p-contained in exosomes of BMMSCs was able to alleviate SCIRI.

## Discussion

Although the role of miR-124-3p has been investigated in traumatic brain injury [16], the specific function of miR-124-3p in SCIRI has not been delineated. This study was designed to understand how miR-124-3p carried in BMMSC-derived exosomes affected SCIRI. We have demonstrated that exosomal miR-124-3p promotes polarization of M2 macrophage by regulating Ern1, therefore alleviating SCIRI.

Ern1 expression in SCIRI was evaluated and found to be highly expressed. Consistently, Ern1 was highly expressed in Alzheimer’s disease induced by neurotoxicity, where inhibiting Ern1 was found beneficial for treatment [17]. Thus, we speculated that silencing Ern1 expression may attenuate nerve injury in SCIRI.

Subsequently, targeting relationship between miR-124-3p and Ern1 was predicted using bioinformatic analysis, which was confirmed by dual-luciferase reporter assay and Pearson’s correlation coefficient. In our study, we confirmed that BMMSC-derived exosomes could transfer miR-124-3p, which then negatively regulated Ern1 expression in macrophages. In a related finding, miRNAs in exosomes secreted by BMMSCs have shown diagnostic and therapeutic properties as they were found to mediate tumor growth, angiogenesis, and metastasis [18]. For example, exosomal miR-124-3p from BMMSCs has been found to alleviate inflammation and oxidative stress injury in vivo [19]. Furthermore, we also found that exosomal miR-124-3p inhibited cell apoptosis in vivo and attenuated tissue impairment in SCIRI. Consistently, the inhibition of neuronal apoptosis is consistent with neuroprotection against SCIRI [20].

Further, we measured polarization of M2 macrophage in SCIRI, as M2 macrophage is reported to be neuroprotective in spinal cord injury [21]. Promoting M2 macrophage polarization has been found to promote repair of tissue and attenuate spinal cord injury [22]. Therefore, a better understanding of the mechanisms improving M2 polarization may be instrumental for advancing SCIRI treatment. We discovered M2 macrophage polarization was lowered in SCIRI accompanied with lower expression of M2 polarization markers Arg1, Ym1, and Fizz. However, upon silencing Ern1 expression, the expression of Arg1, Ym1, and Fizz was upregulated, indicating that Ern1 was negatively correlated with M2 polarization. Consistent with our work, Ern1 regulating M2 macrophage polarization was also reported before, in which Ern1 affected M2 polarization in a cell-autonomous fashion to reduce proinflammatory cytokines in obesity [23].

## Conclusion

**Fig. 9:**
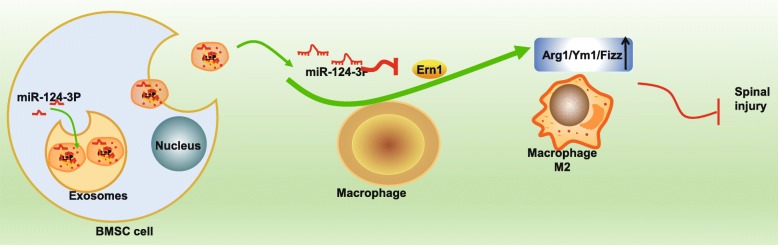
Schematic image of potential molecular mechanisms. Exosomes carrying miR-124-3p derived from BMMSCs enhance M2 macrophage polarization by regulating Ern1, thus alleviating spinal injury in SCIRI. In SCIRI rats, exosomes were derived from BMMSCs expressing miR-124-3p, which elevated Arg1, Ym1, and Fizz expression and promoted M2 macrophage polarization by inhibiting Ern1 expression. Furthermore, exosomal miR-124-3p suppressed cell apoptosis and nerve injury induced by SCIRI

Taken together, exosomal miR-124-3p from BMMSCs negatively regulated Ern1, thus promoting M2 macrophage polarization and alleviating SCIRI. Evaluation of hindlimb motor function and BSCB integrity in SCIRI rats provided confirmatory evidence that miR-124-3p ameliorated the nerve injury caused by SCIRI. Furthermore, upon injection of exosomal miR-124-3p, SCIRI-induced cell apoptosis was found inhibited (Fig. 9). Thus, miR-124-3p may be considered a novel therapeutic target for SCIRI by inhibiting Ern1 expression and augmenting M2 polarization. Our study serves to improve the molecular understanding of SCIRI and proposes a potential target for prevention of nerve damage in SCIRI. However, further investigations are warranted to clarify how Ern1 may regulate macrophage polarization and whether other miRNAs can regulate Ern1 in SCIRI. These findings also suggest that miR-124-3p-based therapeutics could bear potential for enhancing therapeutic outcomes in other diseases besides SCIRI that are associated with nerve injury.

## Data Availability

The datasets generated/analyzed during the current study are available.
